# Supplementary material for: Factors determining antibiotic use in the general population: A qualitative study in Spain
Source: PLoS One. 2021 Feb 4;16(2):e0246506. doi: 10.1371/journal.pone.0246506 (PMC7861377; doi:10.1371/journal.pone.0246506)
Supplement: S1 File — (DOCX) [file pone.0246506.s002.docx]

**Script of the focus group sessions** *(Knowledge and attitudes about resistance to antibiotics)*

**Remember:**

*- Brief introduction of the study and its objectives*

*- Summary of group norms (all opinions are valid, order and confidentiality)*

**Start-up of the focal group.**

***Factors exploration:***

**1. Knowledge, attitudes and behaviours**

- Recently, several news about the use of antibiotics has been published in the press, an example is this news published in *“El Correo Gallego”*, which says that Galicia is one of the communities where more antibiotics are consumed -***"Galicia leads the consumption of antibiotics after a rebound of 37.84% in the dose per inhabitant."***- What would explain this? Is this situation worrying? <http://www.elcorreogallego.es/galicia/ecg/galicia-encabeza-consumo-antibioticos-un-repunte-37-84-dosis-habitante/idEdicion-2017-03-09/idNoticia-1044993/> **knowledge about antibiotics and bacterias*

- What symptoms do you think tell us we need antibiotics? **** knowledge of pathology***

- Recent studies focused in doctors and pharmacists reveal that they consider that patients are pressuring them to get antibiotics. Do you think that in some cases this is so? Why do you think that people can put pressure on the doctor or pharmacist? **** pressure***

- Do you think that after going to the consultation people have doubts? why? **** doctor-pacient relationship***

+ Do you think that people take the treatment properly? (complete pattern, leave it when you are well, add other AB ...) **** adherence***

+ Do you think there are other ways to get AB without having to go through the doctor? why? **** access / other ways***

+ Do you think that people find it difficult to go to the doctor and therefore look for other alternatives? **** Access***

+ Do you think that there are people who use the antibiotics that were left over after being sick, in case they are again? **** other ways***

**2. Antibiotic resistances**

- Do you hate to talk about resistance to antibiotics? Where do you think the information comes from?

- Do you think that the population has the necessary knowledge about the use of antibiotics? ****consequences***

**3. Perceived magnitude of the problem**

- Do you think people find it a worrisome problem? Current or future?

- After all the comments, do you think there is someone who causes this? (doctors who prescribe more, pharmacies where to get AB without prescription) **** guilt attribution***

- How do you think the use of antibiotics could be improved?
