# Supplementary material for: Factors determining antibiotic use in the general population: A qualitative study in Spain
Source: PLoS One. 2021 Feb 4;16(2):e0246506. doi: 10.1371/journal.pone.0246506 (PMC7861377; doi:10.1371/journal.pone.0246506)
Supplement: Attachment — Submitted filename: Response to reviewer.docx [file pone.0246506.s004.docx]

Reviewer's Responses to Questions

**Comments to the Author**

1. If the authors have adequately addressed your comments raised in a previous round of review and you feel that this manuscript is now acceptable for publication, you may indicate that here to bypass the “Comments to the Author” section, enter your conflict of interest statement in the “Confidential to Editor” section, and submit your "Accept" recommendation.

Reviewer #1: All comments have been addressed

Reviewer #2: (No Response)

2. Is the manuscript technically sound, and do the data support the conclusions?

The manuscript must describe a technically sound piece of scientific research with data that supports the conclusions. Experiments must have been conducted rigorously, with appropriate controls, replication, and sample sizes. The conclusions must be drawn appropriately based on the data presented.

Reviewer #1: Yes

Reviewer #2: Partly

3. Has the statistical analysis been performed appropriately and rigorously?

Reviewer #1: N/A

Reviewer #2: Yes

4. Have the authors made all data underlying the findings in their manuscript fully available?

The [PLOS Data policy](http://www.plosone.org/static/policies.action#sharing) requires authors to make all data underlying the findings described in their manuscript fully available without restriction, with rare exception (please refer to the Data Availability Statement in the manuscript PDF file). The data should be provided as part of the manuscript or its supporting information, or deposited to a public repository. For example, in addition to summary statistics, the data points behind means, medians and variance measures should be available. If there are restrictions on publicly sharing data—e.g. participant privacy or use of data from a third party—those must be specified.

Reviewer #1: Yes

Reviewer #2: Yes

5. Is the manuscript presented in an intelligible fashion and written in standard English?

PLOS ONE does not copyedit accepted manuscripts, so the language in submitted articles must be clear, correct, and unambiguous. Any typographical or grammatical errors should be corrected at revision, so please note any specific errors here.

Reviewer #1: Yes

Reviewer #2: No

Thank you for your suggestion. We have submitted the article for review by a native English speaker

6. Review Comments to the Author

Please use the space provided to explain your answers to the questions above. You may also include additional comments for the author, including concerns about dual publication, research ethics, or publication ethics. (Please upload your review as an attachment if it exceeds 20,000 characters)

Reviewer #1: All comments addressed to the extent possible. The manuscript is technically sound, and the data support the conclusions. Recommend to accept manuscript for publication.

Reviewer #2: 1) The most important revision is to get a very careful english proofreading

Thank you for your suggestion. We have submitted the article for review by a native English speaker
2) In the abstract, the current conclusion is unclear and is not from the key findings.

Thank you, We have changed the conclusions of the abstract in order to be more clear

3) The reasons why authors chose over and under 65 years old were not explained well.

Thank you, we have included a new explanation in page 3 line 80-83:

*We decided to made this two groups to better explore the differences in the acces to the heathcare facilities (assuming more time in retirees), and also to explore the differences in the relationship with the doctor between older and yougers.*

4) Each quote shall include participant's age. (eg, 45yo W)

Thank you for your suggestion

We have to apologize but we dont have this information available. We have the range of age of each group but we dont have the exact age of each participant. We have included the group age in order to make easier the reading.

5) Add how the transcription and translation were conducted.

Thank you for your apreciation. We have included how transcription were conducted in page 4 line 109-112. The transcripts were first analyzed to obtain the results of the study. Subsequently, those that best represented the results were identified and selected. Only these quotes were sent for translation by a native English speaker.

7. PLOS authors have the option to publish the peer review history of their article ([what does this mean?](https://journals.plos.org/plosone/s/editorial-and-peer-review-process#loc-peer-review-history)). If published, this will include your full peer review and any attached files.


If you choose “no”, your identity will remain anonymous but your review may still be made public.


**Do you want your identity to be public for this peer review?** For information about this choice, including consent withdrawal, please see our [Privacy Policy](https://www.plos.org/privacy-policy).

Reviewer #1: **Yes:** Elsy Ramia, PharmD, MPH, BCPS

Reviewer #2: No

[NOTE: If reviewer comments were submitted as an attachment file, they will be attached to this email and accessible via the submission site. Please log into your account, locate the manuscript record, and check for the action link "View Attachments". If this link does not appear, there are no attachment files.]
